# Estimating soil erosion utilizing geospatial method and revised universal soil loss equation (RUSLE) of Abu Ghraibat Watershed, Eastern Misan Governorate, Iraq

**DOI:** 10.1038/s41598-025-33403-x

**Published:** 2025-12-24

**Authors:** Bashar F. Maaroof, Hashim H. Kareem, Jaffar H. Al-Zubaydi, Nadhir Al-Ansari, Mohamed Alkhuzamy Aziz, Dhia Alden A. AL-Quraishy, Ban AL-Hasani, Mawada Abdellatif, Iacopo Carnacina, Rayan G. Thannoun, Manal Sh. Al-Kubaisi, Sama S. Al-Maarofi

**Affiliations:** 1 Department of Geography, University of Babylon, Hillah, 51001 Babil Iraq; 2 Department of General Sciences, University of Misan, Amarah, 62001 Misan Iraq; 3 Department of Applied Geology, University of Babylon, Hillah, 51001 Babil Iraq; 4 Department of Civil, Environmental, and Natural Resources Engineering, Lulea University of Technology, Lulea, 97187 Sweden; 5 Department of Geography, University of Fayoum, Fayoum, 63511 Egypt; 6 Department of Geography, University of Wasit, Wasit, 52001 Iraq; 7 Faculty of Engineering Technology, Civil Engineering and Built Environment Department, Liverpool John Moores University, Liverpool, L3 5UX UK; 8 Remote Sensing Center, University of Mosul, Mosul, 6231 AZ Iraq; 9 Department of Geology, University of Baghdad, Baghdad, 10001 Iraq; 10 Faculty of Science, Department of Environmental Sustainability, Lakehead University, 500 University Avenue, Orillia, ON L3V 0B9 Canada

**Keywords:** Geohazards, Soil degradation, GIS, RUSLE, Abu ghraibat watershed, Climate sciences, Ecology, Ecology, Environmental sciences, Natural hazards

## Abstract

This study examined the synergistic and independent effects of soil properties, vegetation cover, conservation practices, and slope on the spatial distribution characteristics of soil erosion in the Abu-Ghraibat watershed in 2024. Soil samples have been collected and analyzed in the laboratory, along with high-resolution satellite imagery, meteorological data, and digital elevation model (DEM) data. The findings indicate that soil erosion in the Abu-Ghraibat watershed in 2024 was minimal, with a progressively increasing severity from north to south. In the studied area, grassland accounts for over 50% of soil erosion, with regions with vegetation coverage > 30% as the primary contributors, all of which are influenced by slope. Moreover, the enhancement of vegetation in the lower strata of the basin and in grasslands, especially on slopes ranging from 10° to 45°, along with the conversion of sloping woodlands and grasslands into terraces, has proven an effective strategy for mitigating soil erosion in the Abu-Ghraibat watershed. The present study has demonstrated that the RUSLEGIS integrated model may serve as an effective instrument for quantitatively and spatially mapping soil erosion at the watershed level in the Abu-Ghraibat, while accounting for the provision of landscape services.

## Introduction

Soil is a natural resource, and anthropogenic and environmental factors have led to its degradation and reduced productivity^1^. Soil degradation is a critical environmental issue primarily linked to socio-economic aspects. All scientific evidence suggests that soil degradation is predominantly caused by human mismanagement of land, while the impact of natural processes (such as climate, geology, and environmental factors) on soil productivity degradation is minimal compared to the effects of human activities^2^. The loss of soil’s ability to provide essential landscape services, including habitats, fertile agricultural soils, and clean water, is one of the most significant consequences of soil degradation. The total area of land affected by soil degradation due to human activities is estimated at 2 billion hectares^3^. Consequently, the land areas impacted by soil degradation from erosion are estimated at 1,100 million hectares due to water erosion and 550 million hectares due to wind erosion^4^. Soil erosion in Iraq has a profound impact on the agricultural sector, siltation in reservoirs, soil degradation, and other aspects of the country. Additionally, it is essential to acknowledge incorrect government policies that neglect necessary intervention measures to conserve water and soil, alongside issues such as population growth, deforestation, and land cover loss^5^. The International Union for Conservation of Nature defines soil degradation as “the deterioration of the natural potential of any soil form that affects the integrity of ecosystems, including a reduction in their sustainable ecological productivity”^6^.

Concerns about soil erosion have recently emerged in the eastern regions of the Misan Governorate, where roughly 57% of the area is experiencing moderate to severe soil erosion. This situation is alarming, as it could worsen soil erosion, negatively impacting food security and agricultural productivity. In the context of land degradation, understanding current soil erosion rates is crucial, especially in areas where mining and agriculture are prevalent^7^. The present study aims to determine the extent, distribution, and type of soil erosion, as well as the primary factors contributing to it. This research can help land-use managers make more informed decisions. It may also promote further investigation into developing practical solutions to mitigate soil erosion in this region. The Chinese Soil Loss Equation (CSLE), the Universal Soil Loss Equation (USLE), the Revised Universal Soil Loss Equation (RUSLE), the Soil and Water Assessment Tool (SWAT), and other models are now considered well-established empirical tools for assessing soil erosion^8,9^.

Several recent studies have examined soil erosion in various locations worldwide^10^. Analyzed soil erosion dynamics by combining terrain characteristics with socioeconomic factors that affect the Loess Plateau^11^. Consider the impact of climate change on soil erosion, accounting for rainfall patterns and changes in land use and land cover (LULC)^12^. Evaluated the annual rates and spatial distribution of soil erosion in the Jamuna Basin using the Revised Universal Soil Loss Equation (RUSLE) model in Bangladesh. The RUSLE model is widely recognized for soil erosion assessment and is an excellent tool for monitoring erosion suitability, yielding highly reliable results^13^. Furthermore, the RUSLE model was adapted for the Abu Ghraibat catchment in southeastern Iraq, yielding a model that enables quantitative assessment of rainfall, vegetation, and soil erosion.

## Materials and methods

### Study site

**Fig. 1:**
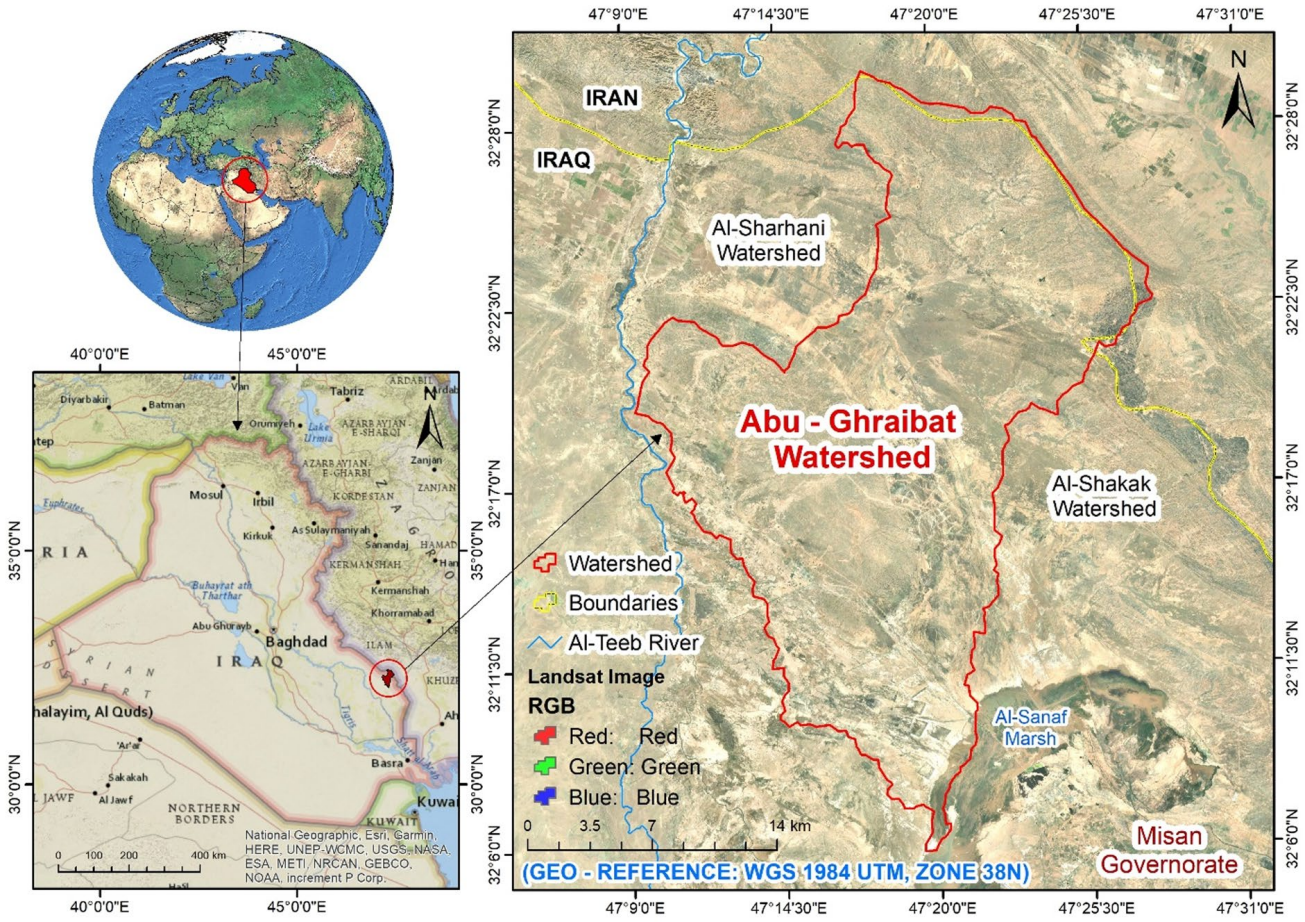
Location of the study area in Iraq.

**Fig. 2:**
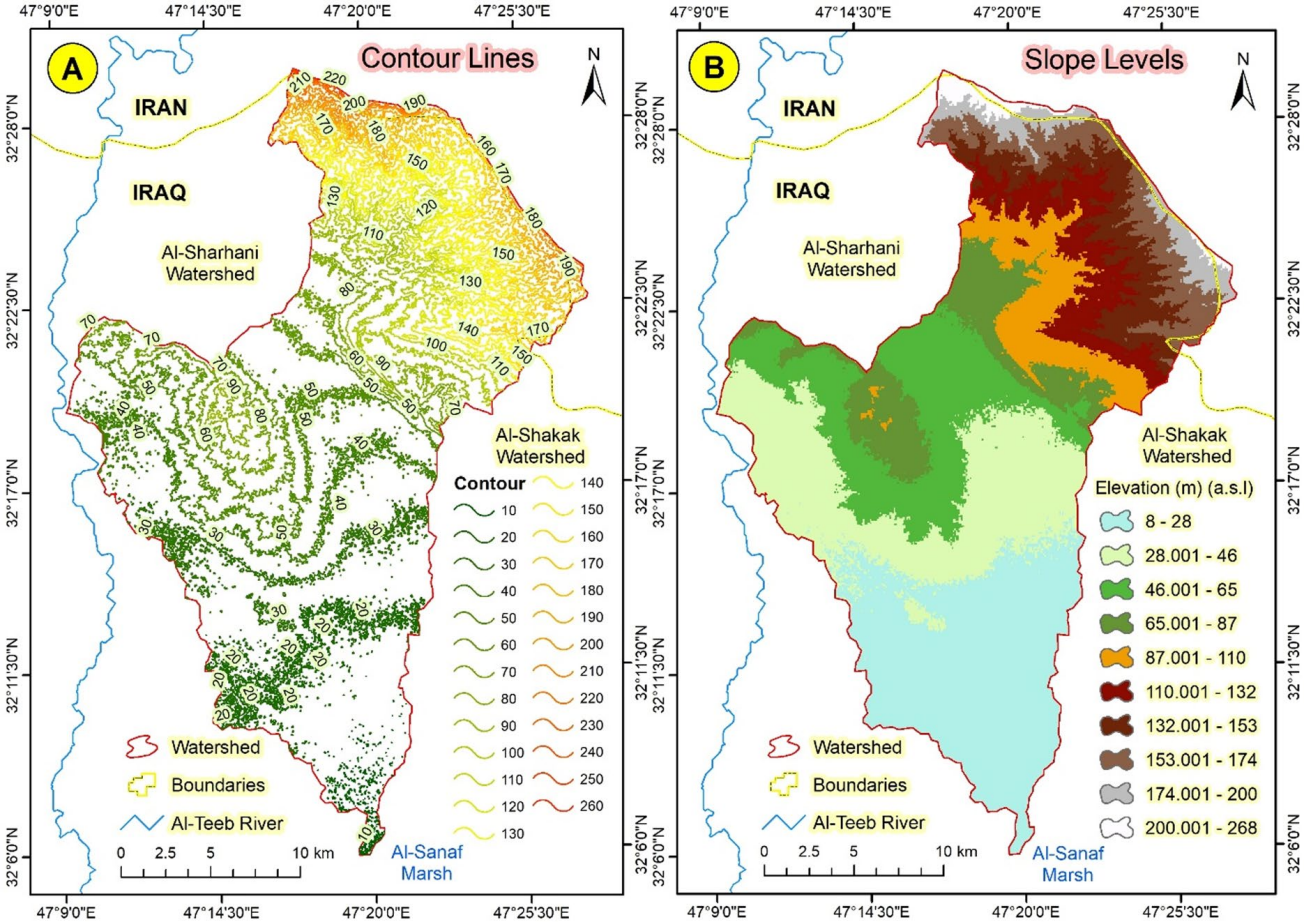
(**A**) Contour lines and (**B**) Slope Levels of Abu Ghraibat watershed.

**Fig. 3:**
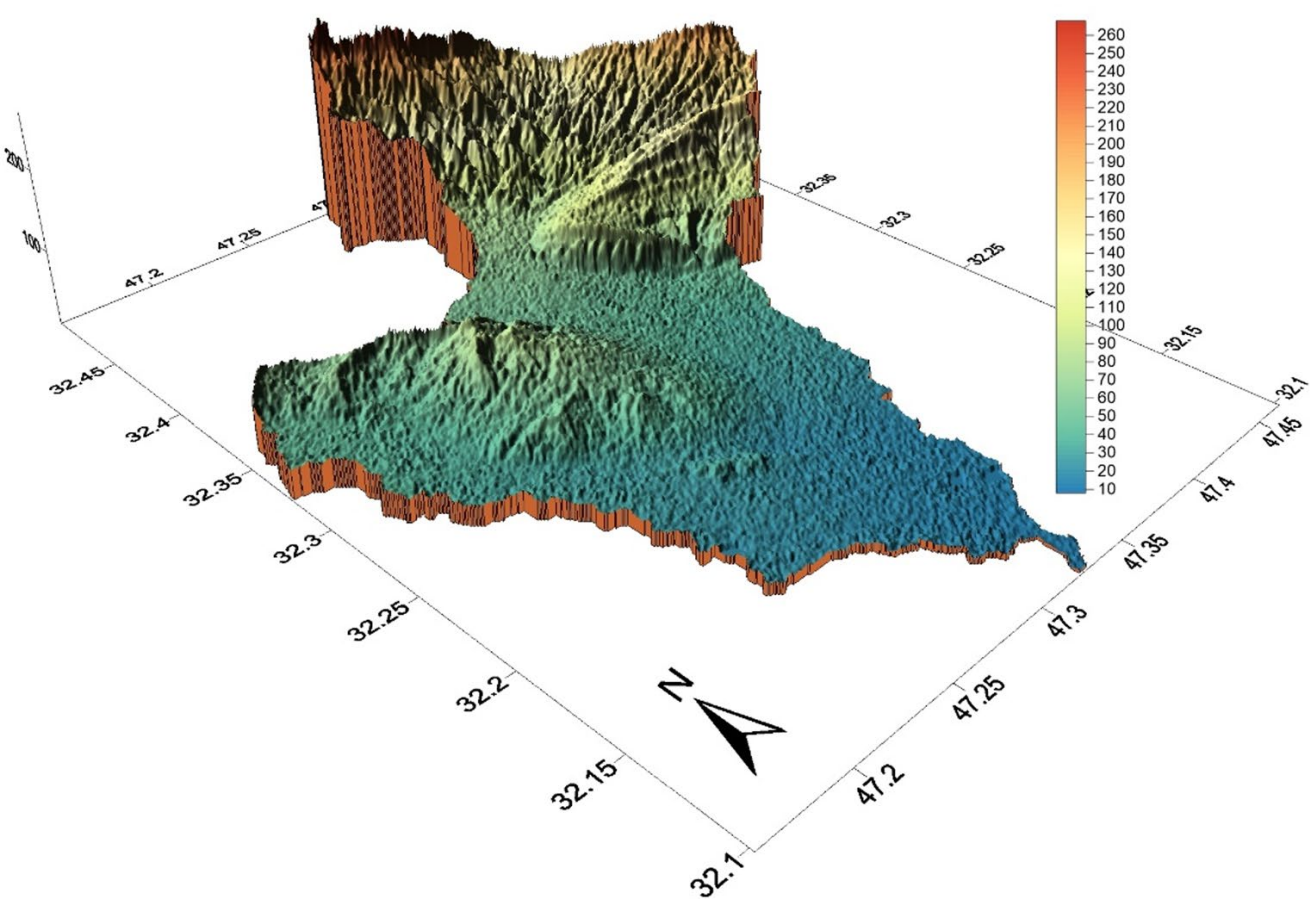
The 3D model of the Abu Ghraibat watershed.

The Abu Ghraibat watershed is situated in the eastern regions of the Misan Governorate in southeastern Iraq, within the Al-Jazeera Eastern Region^14^. Consequently, the study area lies within the Mesopotamian plain, which is characterized by its rich sediments resulting from the floods of the Tigris and Euphrates rivers^15^. Geographically, the Abu Ghraibat watershed is bordered by Iranian territory to the north and northeast, to the east by the Al-Shakak watershed, to the south by the Al-Sanaf Marsh, and to the west by the Al-Teeb River^16^. Astronomically, the Abu Ghraibat watershed is positioned between longitudes (47°09′18.773″E − 47°27′57.589″E) east and latitudes (32°05′50.956″*N* − 32°29′39.368″N) north (Fig. 1). Its area covers 554.751 km² and its perimeter is 142.480 km. The total length of the Abu Ghraibat watershed is 45.295 km, extending from its sources in the northern regions near the Iraqi-Iranian border to its outlet in the Al-Sanaf Marsh in the south. The watershed slopes from the northeast towards the south and southwest, and is currently arid^17^. Water flows into it following rainfall in irregular torrents, shaping its hydro-geomorphological characteristics^18^. The highest point in the watershed is 220 m (a.s.l.), while the lowest point is 10 m (a.s.l.) (Figs. 2 and 3).

**Fig. 4:**
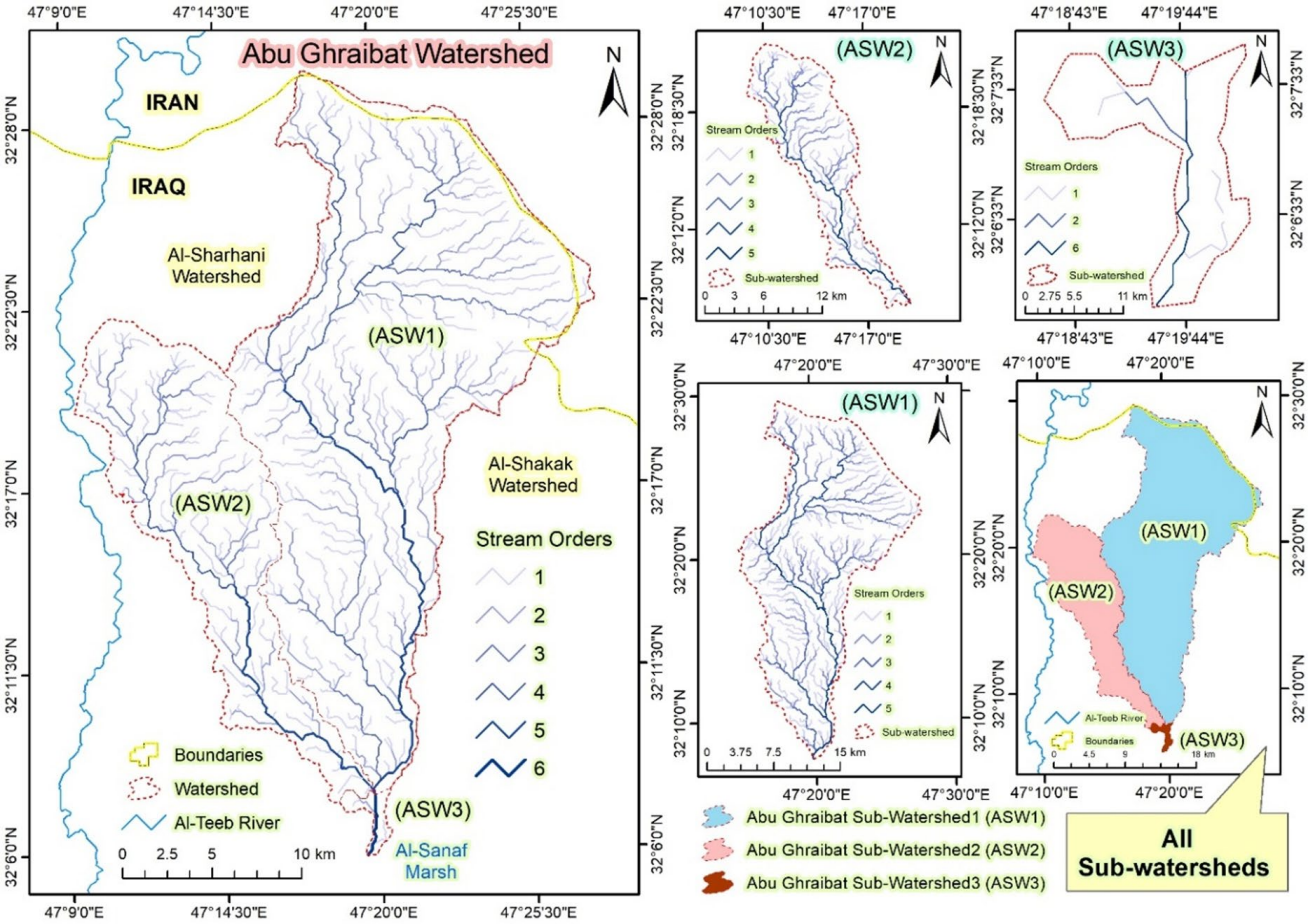
The drainage basin network of Abu Ghraibat watershed and its sub-watersheds.

The Abu Ghraibat watershed is divided into three sub-watersheds:

1. **Abu Ghraibat sub-watershed 1 (ASW1)**: This sub-watershed is situated in the eastern part of the study area. It covers an area of 399.893 km², has a perimeter of 117.093 km, and is 38.671 km long (Table 1; Fig. 4).

**Table 1:** The area, perimeter, and length of Abu Ghraibat sub-watersheds.

Sub-watersheds	Area (km^2^)	Perimeter (km)	Length (km)
Abu Ghraibat sub-watershed (1) (ASW1)	399.893	117.093	38.671
Abu Ghraibat sub-watershed (2) (ASW2)	149.128	83.831	31.054
Abu Ghraibat sub-watershed (3) (ASW3)	4.433	13.834	5.023

2. **Abu Ghraibat sub-watershed 2 (ASW2)**: This sub-watershed is located in the western part of the study area. It spans an area of 149.128 km², has a perimeter of 83.831 km, and is 31.054 km long (Table 1; Fig. 4).
3. **Abu Ghraibat sub-watershed 3 (ASW3)**: This sub-watershed is located in the southern part of the study area. It encompasses an area of 4.433 km², has a perimeter of 13.834 km, and is 5.023 km long (Table 1; Fig. 4).

**Fig. 5:**
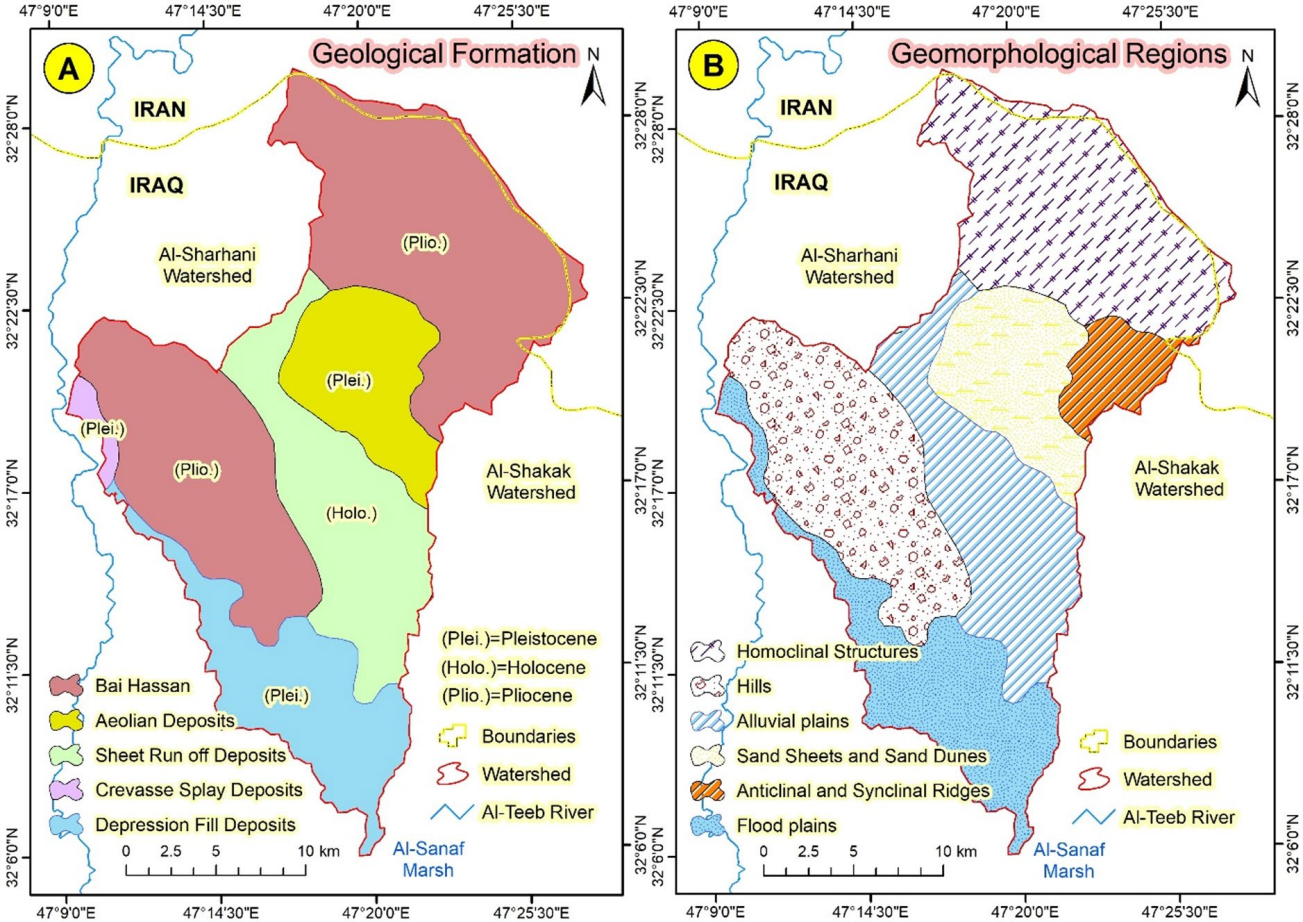
(**A**) Geological formation and (**B**) Geomorphological regions of Abu Ghraibat.

Geologically, the Abu Ghraibat watershed is situated in the southeastern region of the Mesopotamian Plain sedimentary basin^19^. This basin receives significant sediment yearly from the Tigris and Euphrates rivers^20^. Various geological formations are distributed throughout the study area. To the north, northeast, and west, one can find the Bai Hassan formations, which contain rock deposits and erosion-formed conglomerates. In the northern section of the study area, this formation appears exposed and somewhat thick (Fig. 5a). The stratigraphic column indicates that clay layers, typically flat and reaching a thickness of 580 m, constitute the upper section. At the same time, the lower part comprises layers of lime. Sheet run-off deposits are scattered across the center of the study area^21,22^. Although these deposits originated early in the Pleistocene epoch, their surface layers date to the Holocene. These deposits arise from alluvial fans laid down in the northern and northeastern areas of the Al-Jazira Eastern Region, situated north of the study area^23^. Aeolian deposits cover an area of 65.71 km² and a longitudinal strip extending up to 13.267 km adjacent to the Bai Hassan Formation. These deposits consist of silt and fine sand, typically reaching heights of 5 m. Wind action has transported fine sediments from the nearby floodplain areas, forming these deposits^24^.

The study area is geomorphologically divided into six topographic regions; each formed during a distinct geological period. One of these regions is the Homoclinal Structure region, formed by regressive erosion processes in areas of rock weakness, such as the edges of faults and the steeply inclined layers in anticlines (Fig. 5b). The hill region extends south of the area above and is higher than the surrounding land. It is characterized by a group of semi-pyramidal or dome-shaped hills, with an average height not exceeding 110 m (a.s.l.) and a moderate slope that helps retain some of the local soils where plants grow during the wet seasons^24^. Rocky ridges and cliffs with steep sides characterize the Anticlinal and Synclinal Ridges region. Tectonic activation processes directly influence this area, resulting in fault ridges that stretch longitudinally throughout the region. To the south of these regions lies the Flood Plains, consisting of flat lands beside river channels, composed of sandy, silty, and clayey debris deposited by rivers during flood events^25^.

**Fig. 6:**
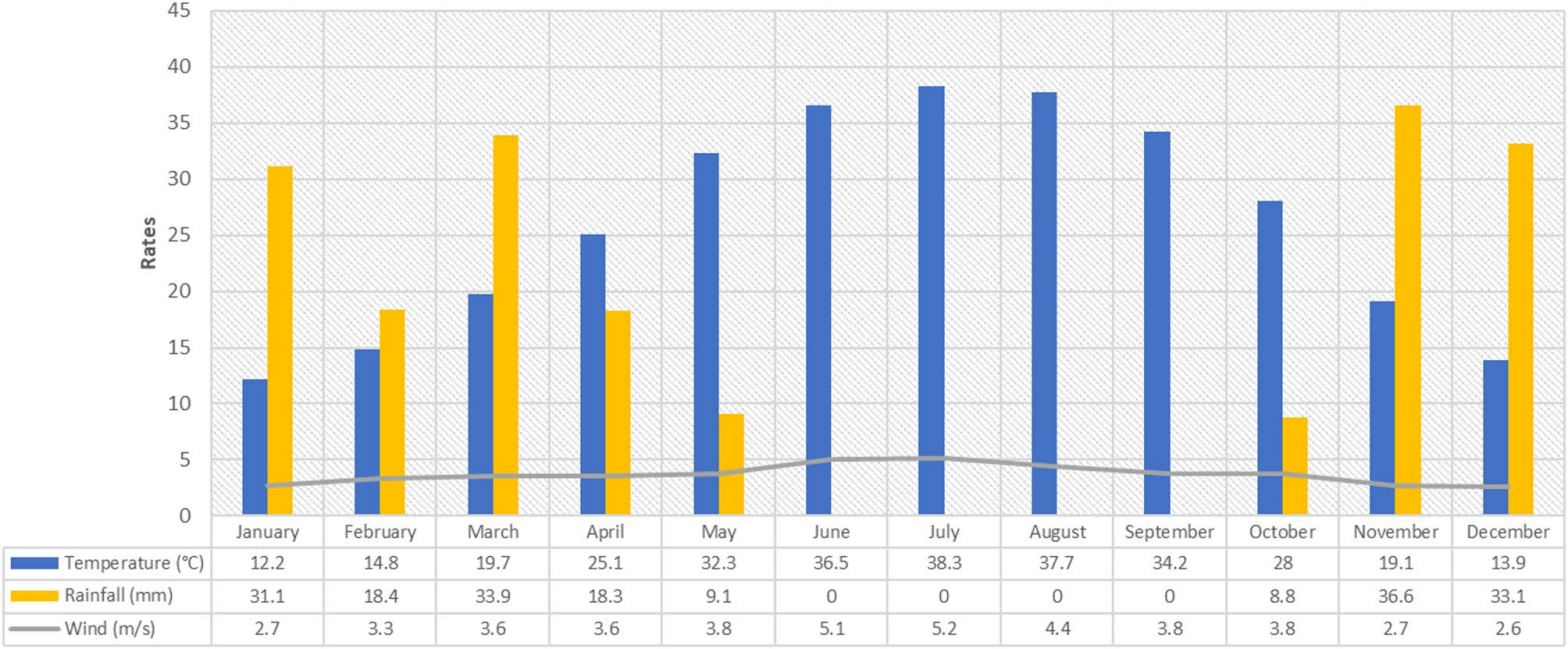
Amarah Climatic Station data for the study area, including temperature, precipitation, and wind speeds, from 1990 to 2020. Iraq | World Meteorological Organization (wmo.int).

Climatically, data from the Al-Amarah climate station for the period 1990 to 2020 were used. This station is located 27 km southwest of the study area and is the closest meteorological station providing continuous records of rainfall and temperatures relevant to the study. Significant seasonal temperature variations characterize the study area^26^. Summer temperatures reach remarkable highs of 32.3, 36.5, and 38.3 °C in June, July, and August, respectively. In winter (December, January, and February), temperatures drop sharply to 13.9, 12.2, and 14.8 °C, respectively. Rainfall occurs from November to May^27^, averaging 9.1 mm. November experiences the highest rainfall, at 36.6 mm. The annual average wind speed is three m/s; in summer, it can reach 5.1, 5.2, and 4.4 m/s, respectively. Figure 6 illustrates that the average wind speed falls to 2.6, 2.7, and 3.3 m/s during winter^28^.

### Soil sampling and laboratory analysis

**Fig. 7:**
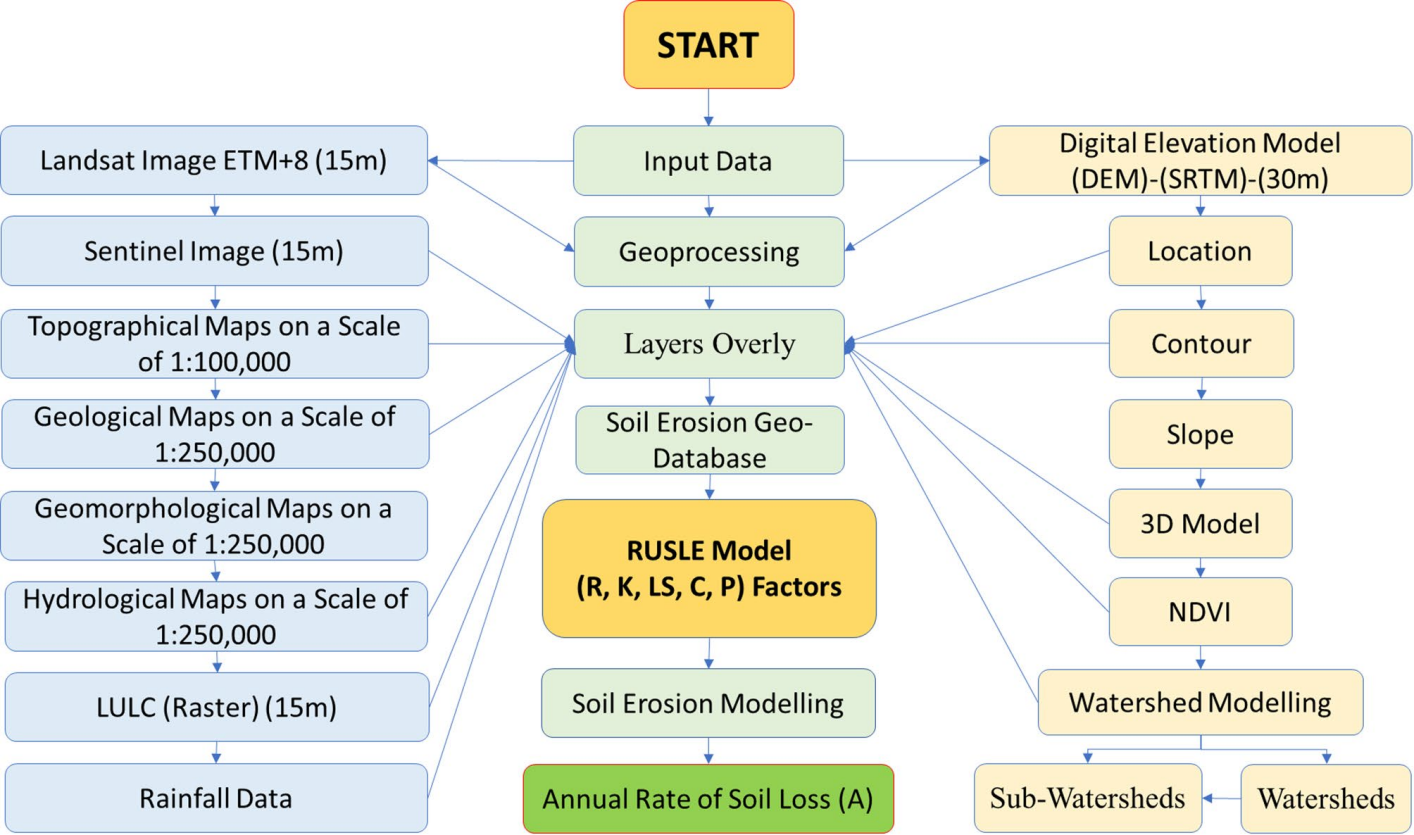
Information flowchart of applied methodology.

A total of 30 surface soil samples (0–20 cm depth) were collected from representative sites across the Abu Ghraibat watershed. The samples, labelled S1–S30, correspond to the locations shown in Fig. 7. Sampling was conducted between March 10 and March 25, 2024, immediately after the main rainfall period, to capture post-erosion surface conditions while minimizing the effects of prolonged drying. During collection, stainless-steel augers and polyethylene containers were used. All tools were cleaned with distilled water between samples to prevent cross-contamination. The collected samples were sealed in airtight plastic bags, labelled, and transported to the Soil and Water Laboratory at the College of Agriculture, University of Misan. In the laboratory, samples were air-dried, sieved (2 mm), and analyzed for key physico-chemical parameters including texture (% sand, silt, clay), bulk density (g cm⁻³), organic matter (%), pH, and electrical conductivity (dS m⁻¹). Analytical procedures followed standard protocols of USDA (2017) and Black (1965). The summary of soil properties is presented in Table 2, where all variables include their respective **measurement units** in the header row. The data were entirely obtained from the present research activities and not from previously published sources.

**Table 2:** Soil properties were used to estimate RUSLE model parameters.

No.	Sub watershed	O.M	SSC	SPC	VFS	Sand	Silt	Clay	Texture	Slope
1	ASW1	0.2	3	4	8	55	40	5	SANDY LOAM	70
2	ASW1	0.1	3	4	8	53	45	2	SANDY LOAM	60
3	ASW1	0.4	3	4	9	52	44	4	SANDY LOAM	50
4	ASW1	0.3	3	4	10	55	43	2	SANDY LOAM	50
5	ASW1	0.4	3	4	11	55	42	3	SANDY LOAM	50
6	ASW1	0.5	3	4	12	53	42	5	SANDY LOAM	40
7	ASW1	0.4	3	4	13	54	42	4	SANDY LOAM	40
8	ASW1	0.4	3	4	13	53	45	2	SANDY LOAM	40
9	ASW1	0.4	3	4	14	51	42	7	LOAM	40
10	ASW1	0.4	3	4	14	51	43	6	SANDY LOAM	40
11	ASW1	0.4	3	4	13	50	43	7	LOAM	40
12	ASW1	0.5	3	4	14	51	42	7	LOAM	38
13	ASW2	0.5	2	4	14	52	40	8	LOAM	38
14	ASW2	0.6	3	4	15	50	41	9	LOAM	38
15	ASW2	0.7	3	4	15	49	41	10	LOAM	30
16	ASW1	0.7	2	4	16	49	43	8	LOAM	30
17	ASW1	0.7	3	4	16	48	41	11	LOAM	30
18	ASW1	0.6	3	4	16	47	40	13	LOAM	30
19	ASW2	0.8	3	5	16	45	40	15	LOAM	25
20	ASW1	0.8	3	5	16	43	43	14	LOAM	25
21	ASW1	0.9	3	5	15	45	43	12	LOAM	20
22	ASW2	0.9	3	5	15	46	42	12	LOAM	20
23	ASW1	0.8	2	5	15	44	41	15	LOAM	20
24	ASW1	0.8	3	5	15	43	43	14	LOAM	20
25	ASW2	0.9	3	5	16	40	44	16	LOAM	20
26	ASW1	0.9	3	5	16	39	43	18	LOAM	20
27	ASW1	1.0	3	5	16	38	44	18	LOAM	20
28	ASW2	1.2	3	6	15	32	35	33	CLAY LOAM	14
29	ASW1	1.4	3	6	15	31	34	35	CLAY LOAM	12
30	ASW3	1.5	3	6	14	29	32	39	CLAY LOAM	10

### Data processing

The soil erosion of the Abu Ghraibat watershed was assessed using geoinformatics applications and the Revised Universal Soil Loss Equation (RUSLE). The geospatial data was sourced from the US Department of Defense’s Digital Elevation Model (DEM) type (SRTM). Along with topographic maps at a scale of 1:100,000 provided by the General Authority for Iraqi Survey, and geological and hydrological maps at a scale of 1:250,000 from the Iraqi Geological Survey, Landsat ETM + 8 satellite imagery for the year 2023, with a spatial resolution of 15 m, was employed. This data was imported into a Geographic Information System (GIS) using ArcGIS V.10.8 and integrated into a topological model as raster layers. In addition to delineating the river drainage network at all orders, the primary and sub-watersheds were identified and produced as vector layers^29^. Various software tools for geographic analysis, including ArcGIS Earth 1.16, Surfer 10, Global Mapper 11, and Google Earth Pro 7.1, were also employed.

**Fig. 8:**
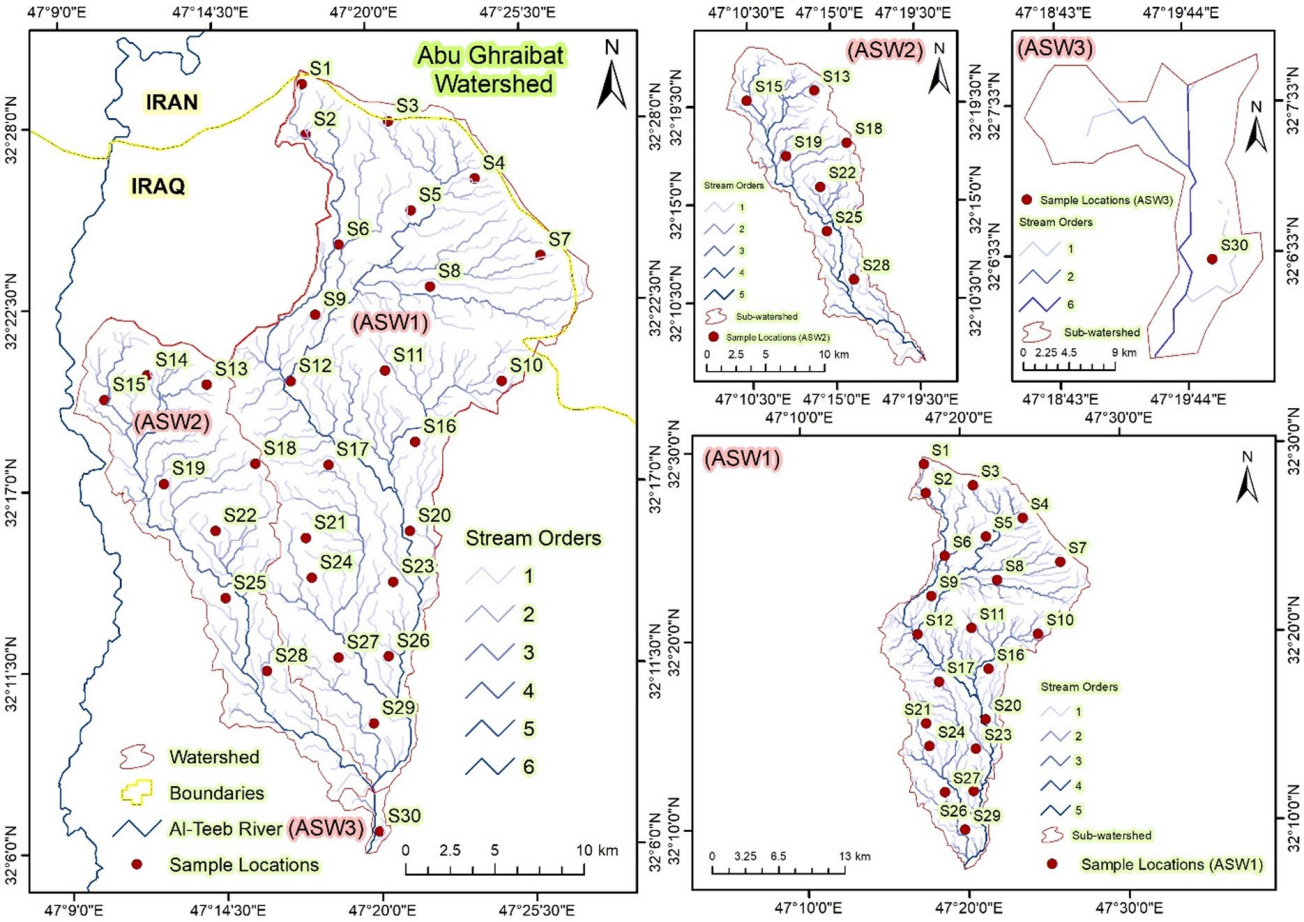
Sample locations of soil in Abu-Ghraibat watershed.

A significant milestone was achieved with the availability of an integrated geospatial database for the study area^30^. This database enabled us to explore the geomorphological aspects of soil erosion within the Abu Ghraibat watershed. We meticulously identified and evaluated environmental factors such as rainfall, terrain, vegetation cover, and soil, and assessed their impact on soil erosion in the study area, all within the framework of geospatial analysis (Fig. 8). The dimensions of soil erosion within the basin, driven by natural processes and forces, were quantified with precision, and the geographic scope of the phenomenon under investigation was clarified through the use of cartographic methods.

The RUSLE model, the most widely used global soil loss predictor, stands out for its simplicity and compatibility with Geographic Information Systems (GIS). Despite being an empirical model, it not only forecasts erosion rates in ungauged watersheds using information on watershed characteristics and local hydroclimatic conditions, but also illustrates the spatial heterogeneity of soil erosion. This practical and cost-effective approach is beneficial in larger areas. By introducing improved methods for calculating soil erosion factors, RUSLE has been extensively utilized to predict average annual soil loss in the Abu Ghraibat watershed. In raster data format, this equation relies on five input factors: soil erodibility, slope length and steepness, cover management, rainfall erosivity, and support practice^31^. Other input variables influence these variables and change over time and space^32^. Therefore, the RUSLE was employed to estimate soil erosion within each pixel. The expression for the RUSLE method is:

1 $$A\,=\,R{\text{ }}*{\text{ }}K{\text{ }}*{\text{ }}LS{\text{ }}*{\text{ }}C{\text{ }}*{\text{ }}P$$

Where A represents the average erosion (ton h-1 y-1), R denotes the rainfall erosivity (MJ mm ha-1 y-1), and K signifies the soil erodibility (ton ha-1 MJ mm). Additionally, LS, C, and P refer to slope length, land cover management, and conservation measures, respectively, and these are dimensionless^32^.

#### Rainfall erosivity factor (R)

The impact of rainfall on topsoil is assessed by rainfall erosivity (R). When raindrops collide with topsoil, they convert kinetic energy into potential energy, creating conditions that encourage soil erosion. As a result, an increase in rainfall intensity corresponds with a rise in rainfall erosivity. This understanding of the relationship between rainfall and soil erosion is crucial in our study^31^. The formula for calculating rainfall erosivity is:

2 $$R\,=\,79\,+\,0.363{\text{ }}*{\text{ }}{P_a}$$

Where: P_a_ is the average annual rainfall.

#### Soil erodibility factor (K)

The K-factor is a quantitative value derived from experiments that indicate the soil’s sensitivity to erosion. It fully expresses the soil’s capacity to erode due to its lack of resistance to runoff and rainfall. In this study, the K-factor was determined using the method proposed by RUSLE, which involves calculating it with the average geometric diameter of soil particles^33^. The precise formula for the calculation is as follows:

3 $$K{\text{ }}={\text{ }}\left[ {2.1{\text{ }}*{\text{ }}{{10}^{ - \,4}}\left( {12{\text{ }}--{\text{ }}OM} \right){\text{ }}{M^{1.14}}+{\text{ }}3.25{\text{ }}\left( {S{\text{ }}--{\text{ }}2} \right)\,+\,2.5{\text{ }}\left( {P{\text{ }}--{\text{ }}3} \right)} \right]{\text{ }}/{\text{ }}100$$

Where: OM = Percentage soil organic matter content, M = (% Silt + % Very Fine Sand) * (100 - % Clay), S = Soil structural code, P = Soil profile permeability rating was obtained using a combination of field observation, and default values were considered for S and P.

#### Slope length factor (LS)

The LS factor summarizes how topography influences soil erosion and significantly impacts soil loss. The local slope gradient affects flow velocity and erosion rate. The slope length indicates the distance between the start and end of the inter-rill processes^34^. The following formula was employed to calculate the LS factor:

4 $$LS{\text{ }}={\text{ }}{\left( {X{\text{ }}/{\text{ }}22.13} \right)^m}\left( {0.065\,+\,0.045{\text{ }}S\,+\,0.0065{\text{ }}{S^2}} \right)$$

Where: S = Slope (%) calculated directly from the DEM, X = Value obtained by multiplying the flow accumulation by the cell value, M = Value that varied from 0.2 to 0.5 depending on the slope. 0.5 for slopes exceeding 5%, 0.4 for slopes 3–5%, 0.3 for 1–3%, and 0.2 for slopes < 1.0%.

#### Cover management factor (C)

The cover management factor C represents the influence of crops and other management practices on erosion rates. Vegetation cover is the second most vital factor in mitigating soil erosion risk, after terrain^35^. Its value ranges from 0 (water bodies) to 1 (barren land), reflecting the absence of vegetation, root biomass, or other surface covers that prevent soil erosion. Ground cover absorbs rainfall, enhancing infiltration and diminishing rainfall energy^36^. The Normalized Difference Vegetation Index (NDVI) was used to determine the cover management factor (C). The following formula was applied to calculate the LS factor:

5 $$C\,=\,0.431\, - \,0.805 * NDVI$$

Where: NDVI = Near-infrared (NIR) – R/ Near-infrared (NIR) + red (R), NIR = Near-infrared band, and R is the red band.

#### Conservation practices factor (P)

The factor of support practices P represents the outcomes of implementing water and soil conservation measures, which reduce the quantity and rate of runoff and the amount of soil loss. A value of 0 signifies no soil erosion in the region, whereas a value of 1 indicates that no conservation measures have been applied^37^.

## Results

### Soil characteristics

Table 2 illustrates nine soil characteristics that directly influence soil erodibility and soil erosivity and thereby determine the RUSLE model’s output. By taking a look at the table, we can notice that the studied soil locations were poor in organic matter, which ranged between 0.1 and 1.5 g.kg, this values actually was lower in the north section in sub watershed (1) (ASW1) and increases gradually towards south section sub watershed (3) (ASW3) which was the higher content in organic matter, the results also showed that the soil structural code (SSC) ranged between (2–3) were the higher values concentrated in the higher elevation sites in SW1 while the lower values were recorded in the low elevation sites in SW3, however, wonderful sand ranged between 8 and 14 gm/kg were the lower quantities were concentrated in the upper positions while the higher values recorded in the lower positions of soil samples, by taking a look on the particle size distribution we can noticed that the percentage soil particles were ranges between (29–55), (32–40), and (5–39) for sand, silt and clay respectively, which produced three groups of soil textures namely (sandy loam, loam and clay loam) as shown in Table (2).

### Factors of RUSLE

#### Rainfall-runoff erosivity (R-factor)

The computed rainfall-runoff erosivity (R-factor) values vary from 323.4935 at the SW1 station to 10.70138 MJ mm ha⁻¹ hr⁻¹ yr⁻¹ at the lower site in the SW3 station (Table 3). The maximum rainfall erosivity value is recorded in the northern region of the Abu Ghraibat watershed, attributed to the elevated terrain, which results in larger drop sizes, comparatively greater precipitation, and steep gradients. Rainfall erosivity progressively diminishes from the watershed’s northern to its southern regions. The south region of the watershed requires soil protection due to higher rainfall than in the north region. The soils in the research area can be classified into three textural groups according to the relative proportions of sand, silt, and clay (Table 3).

**Table 3:** RUSLE model estimated parameters.

No.	Sub watershed	*R*	K	L.S	C	*P*	A
1	ASW1	323.49	0.06	0.13	0.326	1	823
2	ASW1	238.41	0.06	0.14	0.326	1	653
3	ASW1	166.32	0.06	0.14	0.323	1	451
4	ASW1	166.32	0.06	0.14	0.309	1	432
5	ASW1	166.32	0.06	0.14	0.309	1	432
6	ASW1	107.21	0.06	0.14	0.295	1	266
7	ASW1	107.21	0.06	0.14	0.271	1	244
8	ASW1	107.21	0.06	0.15	0.260	1	251
9	ASW1	107.21	0.06	0.14	0.257	1	231
10	ASW1	107.21	0.06	0.15	0.253	1	244
11	ASW1	107.21	0.06	0.14	0.236	1	213
12	ASW1	96.95	0.06	0.14	0.226	1	184
13	ASW2	96.95	0.06	0.14	0.222	1	181
14	ASW2	96.95	0.06	0.14	0.198	1	161
15	ASW2	61.08	0.06	0.14	0.187	1	96
16	ASW1	61.08	0.06	0.15	0.184	1	101
17	ASW1	61.08	0.06	0.14	0.180	1	92
18	ASW1	61.08	0.06	0.14	0.167	1	86
19	ASW2	42.88	0.08	0.14	0.156	1	75
20	ASW1	42.88	0.08	0.15	0.149	1	77
21	ASW1	27.92	0.08	0.14	0.146	1	46
22	ASW2	27.92	0.08	0.14	0.121	1	38
23	ASW1	27.92	0.08	0.14	0.114	1	36
24	ASW1	27.92	0.08	0.14	0.111	1	35
25	ASW2	27.92	0.08	0.15	0.097	1	32
26	ASW1	27.92	0.08	0.14	0.087	1	27
27	ASW1	27.92	0.08	0.15	0.763	1	256
28	ASW2	14.23	0.11	0.12	0.076	1	14
29	ASW1	10.70	0.11	0.12	0.076	1	11
30	ASW3	10.49	0.11	0.11	0.074	1	11

#### Soil erodibility (K-factor)

The determined soil erodibility (K-factor) values varied from 0.058767 to 0.10858 MJ mm h⁻¹ ha⁻¹ yr⁻¹.

#### The topographic factor (L.S)

The topographic factor (L.S) denotes the impact of slope length and steepness on the erosion process. The LS factor was computed using flow accumulation and slope percentage as inputs. The results indicate that the topographic factor value escalates from 0.108 to 0.127 as flow accumulation and slope rise.

#### Crop management factor (C)

The crop management factor (C) ranged between 0.074 and 0.326, with lower values concentrated in the lower regions of sub-watershed 3. In comparison, the higher values were recorded in the upper areas of the sub-watershed.

### Computed Spatial and Temporal average soil loss per unit area (A)

Table 1 shows that the computed spatial and temporal average soil loss per unit area (A) values ranged from 11 to 823 t/yr. The higher quantity of soil loss was recorded in the upper regions of the Abu-Ghraibat watershed. In contrast, lower soil loss was recorded in the lower areas of the watershed.

## Discussion

### Soil properties used in Estimation of RUSLE model parameters

#### Organic matter (OM)

**Fig. 9:**
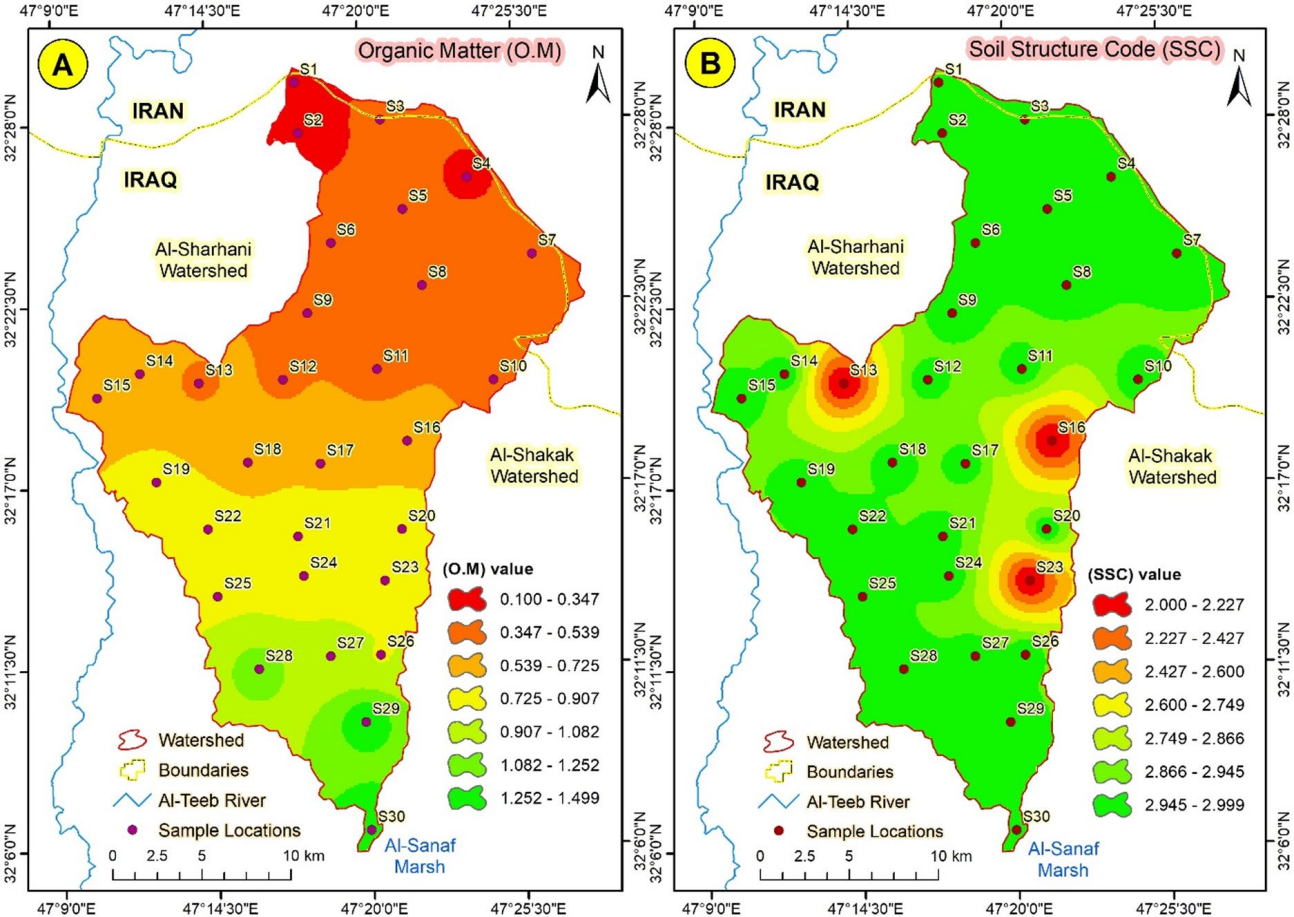
(**A**) Soil organic matter distribution, and (**B**) Soil structure code distribution in Abu-Ghraibat watershed.

Variation in the organic matter content is apparent, as shown in Fig. 1, where its deficiency can be recognized. The soil in the watershed is impoverished in organic matter due to lower vegetation cover, which has already been affected by water scarcity. Organic matter is a crucial factor in enhancing soil structure by forming peds that connect soil particles and prevent them from dispersing and becoming easily eroded. Many researchers have explained how soil organic matter can enhance soil structure and reduce soil erosion hazards^38^. The organic matter content is classified into seven categories, ranging from 0.029 to 0.313 for the first category and from 1.470 to 1.795 for the seventh category; the variation in organic matter content is reflected in the RUSLE parameters and erosion intensity (Fig. 9a).

#### Soil structure code (SSC)

The soil structure code has a strong relationship with organic matter; the higher the number, the better the soil structure. Therefore, the upper sample locations are characterized by similarities in soil structure based on the porosity of organic matter^39^. The low clay content effectively influences soil structure, as shown in Table 2. The soil structure code values ranged from 2 to 3, with no discernible harmonic trend. Figure 9b illustrates the distribution of the soil structure code.

#### Soil permeability code (SPC)

**Fig. 10:**
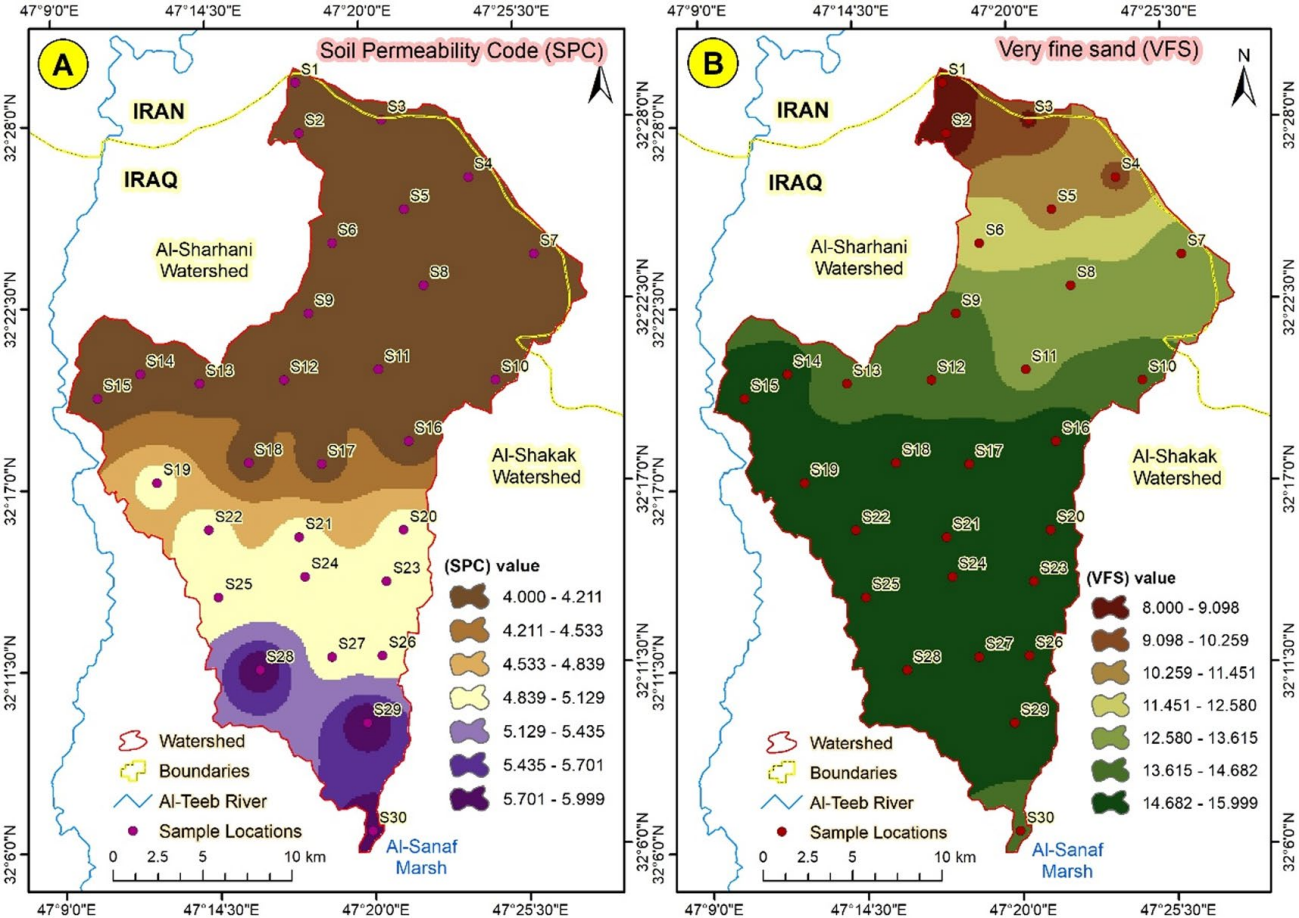
(**A**) Soil permeability code distribution, and (**B**) Very fine sand distribution in Abu-.

The Soil Permeability Code (SPC) indicates the ability of water to penetrate soil layers. This process prevents water runoff, which is considered the most significant factor promoting erosion^40^. The SPC values ranged from 4 to 6, with the higher values concentrated in the lower zones. In contrast, as expected based on the particle size distribution, lower values are found in the upper zones, where fine particles are predominant, and in the lower zones, where coarse particles are more prevalent, as shown in Fig. 10a.

#### Very fine sand (VFS)

The quantity of very fine sand varies according to the location of the samples, with lower values concentrated in the upper locations and higher values found in the lower places. This is expected due to the movement of fine particles from higher to lower areas, caused by gravity and facilitated by wind or water^41^. Figure 10b illustrates the distribution of very fine sand, categorizing this material into seven distinct groups.

#### Particle size distribution

**Fig. 11:**
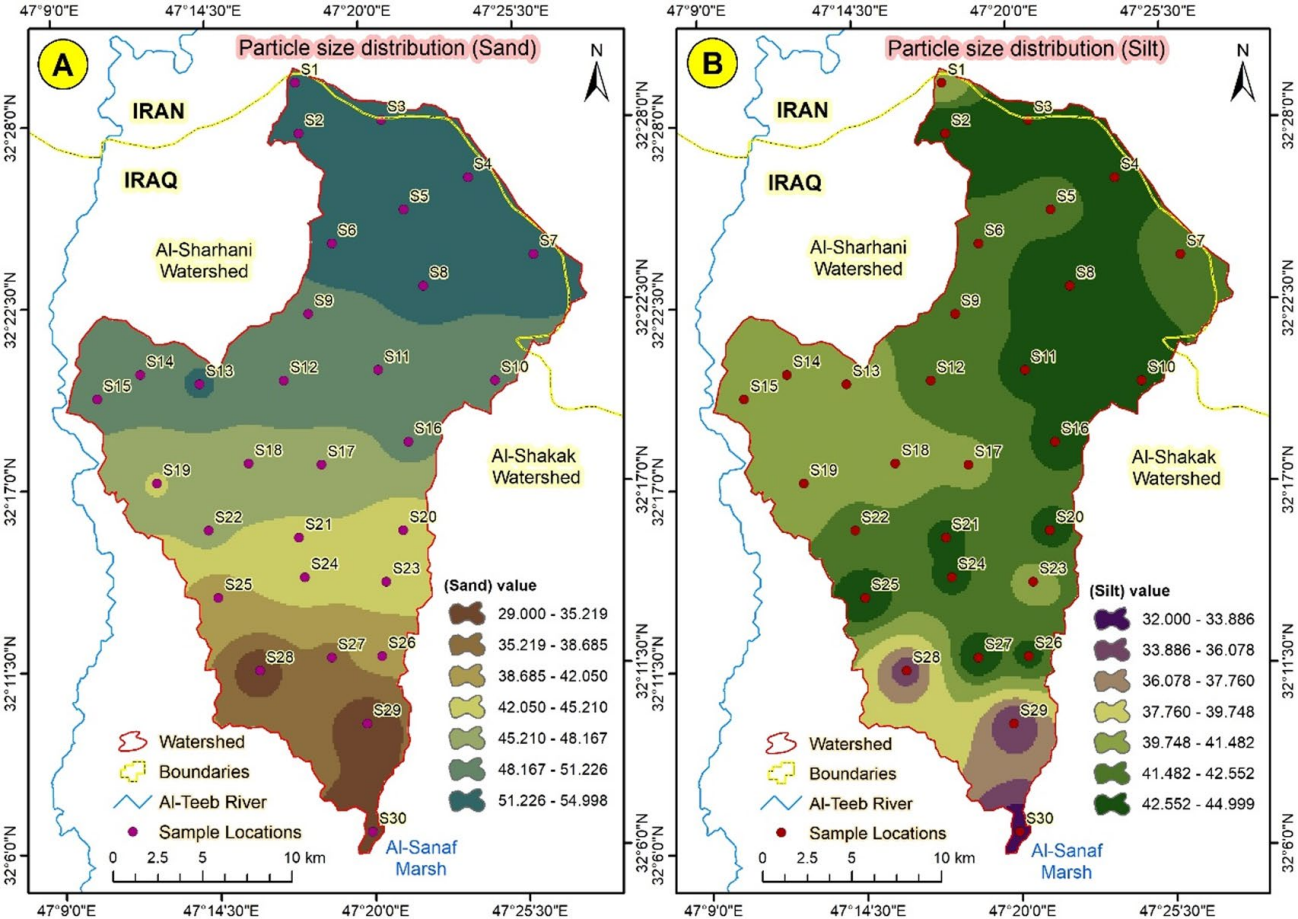
(**A**) Sand fraction distribution, and (**B**) Silt distribution in Abu-Ghraibat watershed.

**Fig. 12:**
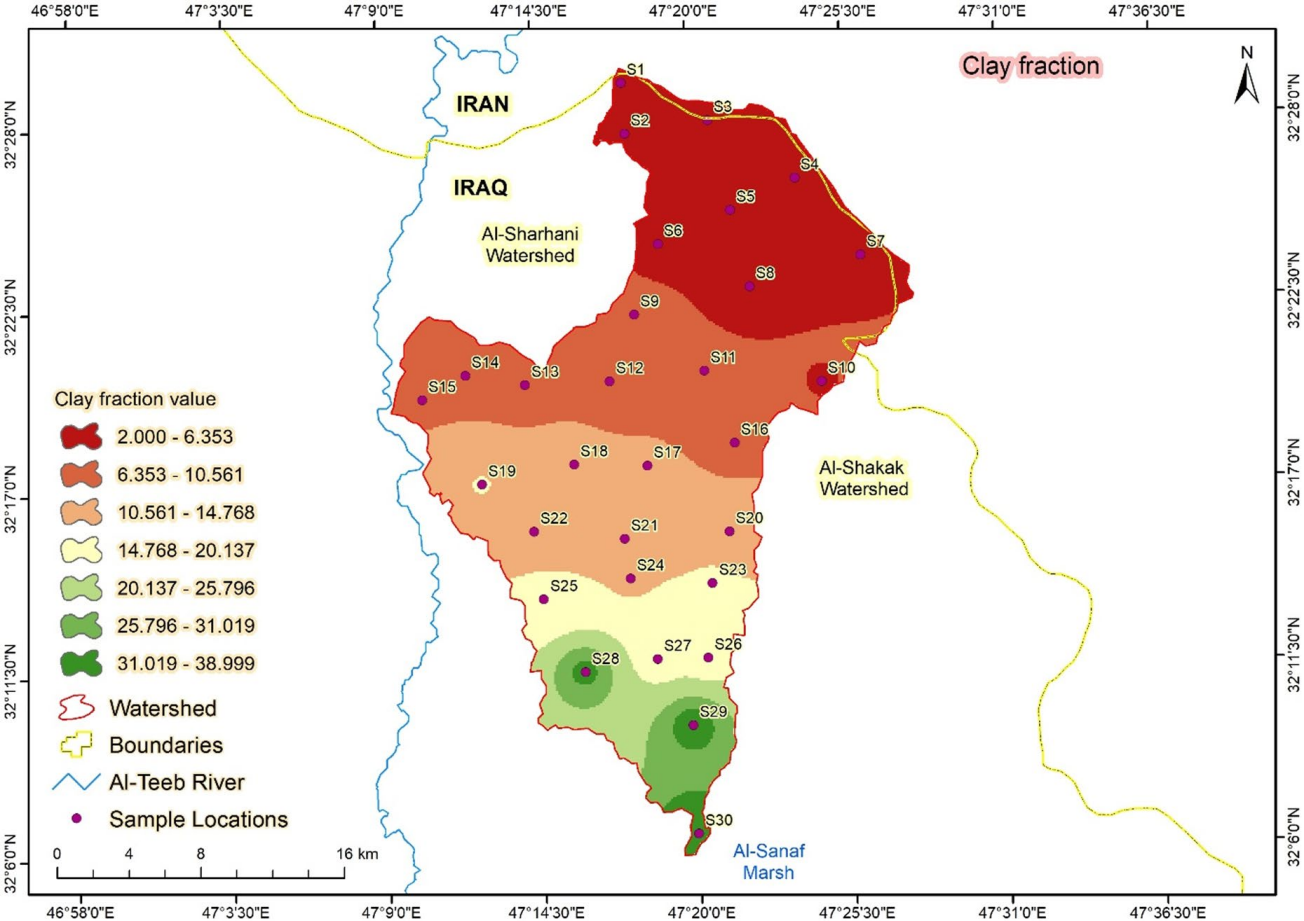
Clay fraction distribution in Abu-Ghraibat watershed.

The percentages of soil fractions varied significantly according to the location of the samples, with the coarse fractions concentrated in the upper locations and the fine particles accumulating in the lower locations^42^. The trend coincided harmoniously with the slope gradient, which determined the sedimentation aspect of soil fractions (Sand, Silt, and Clay) (Figs. 11 and 12). The relationship between soil properties and computed soil loss (A) indicates a strong dependence on both organic matter content (OM) and soil texture, particularly sand percentage (SSC). As shown in Table 2; Fig. 9a, **sample S1**, which has the lowest OM (0.41%) and the highest sand content (78%), recorded the most significant soil loss (31.5 t ha^-1^yr^**-1**^). The low OM reduces aggregate stability and infiltration capacity, enhancing detachment and runoff generation. Conversely, **sample S4**, characterized by relatively higher OM (1.25%) and lower sand content (52%), showed a much lower soil loss (7.8 t ha^**-1**^ yr^-1^), confirming that higher OM enhances soil cohesion and resistance to erosion. These findings demonstrate that soils with **coarser textures and depleted organic matter** are more vulnerable to erosion, consistent with the sensitivity of the RUSLE factor to the cover management (C) and soil erodibility (K) parameters. This quantitative relation strengthens the model’s reliability and agrees with results from semi-arid catchments reported by^43,44^.

The spatial variation of soil loss across the Abu Ghraibat watershed reflects the combined influence of soil properties, topography, and land cover. Soils characterized by high sand content and low organic matter (e.g., S3, S6, and S9) exhibited the highest soil loss rates, as their coarse texture reduces aggregate stability and infiltration capacity, increasing surface runoff and detachment potential. Conversely, areas with higher clay and silt content (S2, S4) showed lower erosion rates due to stronger aggregate cohesion and enhanced water retention. Bulk density was another influential factor; higher bulk density values (> 1.6 g cm⁻³) in compacted or degraded soils were associated with reduced infiltration and increased overland flow, amplifying erosion intensity. In contrast, soils with higher organic matter showed lower soil loss, confirming the stabilizing role of organic matter in improving structure and reducing crust formation. The slope gradient (LS factor) showed a direct relationship with predicted erosion: steeper areas (> 10%) experienced substantially higher soil loss than nearly flat terrain (< 2%), consistent with RUSLE’s sensitivity to slope length and steepness. Similarly, the rainfall erosivity factor (R) showed clear spatial correspondence with erosion-prone zones, particularly in the northeastern sub-catchments receiving higher seasonal rainfall. The combined effects of these parameters explain the observed soil loss patterns illustrated in Fig. 9. These findings align with previous studies in semi-arid regions, confirming that low vegetation cover, high rainfall intensity, and fragile soil structure are the dominant drivers of erosion in similar environments.

Overall, the analysis highlights the strong coupling between soil texture, organic matter, and topographic factors in determining erosion susceptibility. Areas characterized by sandy loam soils and steep slopes should be prioritized for soil conservation practices such as contour ploughing, vegetative barriers, and controlled grazing. The relationship between the computed soil loss (A) and both slope per cent (SPC) and vegetation/soil factor (VSF) is shown in Fig. 10. A clear trend is observed: areas with **steeper slopes (high SPC)** and **low vegetation coverage (low VSF)** exhibit higher soil loss rates. For instance, **samples S1 and S2**, located on slopes exceeding **9–11%**, recorded A values of 31.5 and 27.4 t ha^-1^ yr^-1^, respectively, coinciding with sparse vegetation cover (VSF = 0.28–0.33). In contrast, **sample S5**, which lies on a gentle slope of **3.5%** and has denser vegetation (VSF = 0.65), shows a markedly lower soil loss of 6.2 t ha^-1^ yr^-1^. These results highlight the combined effect of topography and vegetation on erosion intensity, consistent with findings by^45^, who reported similar trends in semi-arid catchments—Figs. 11 and 12 further support this pattern by illustrating the role of **particle size distribution**. Samples with a higher proportion of **coarse sand and silt** (e.g., S1–S2) are more erosion-prone due to reduced aggregate stability and infiltration, while those with **finer textures** (e.g., S4–S5) show greater resistance to detachment and lower A values. Overall, integrating slope gradient, vegetation cover, and soil texture provides a consistent explanation for the spatial variability in soil loss predicted by the RUSLE model in the Abu Ghraibat watershed.

### Factors of RUSLE

#### Rainfall-runoff erosivity (R-factor)

**Fig. 13:**
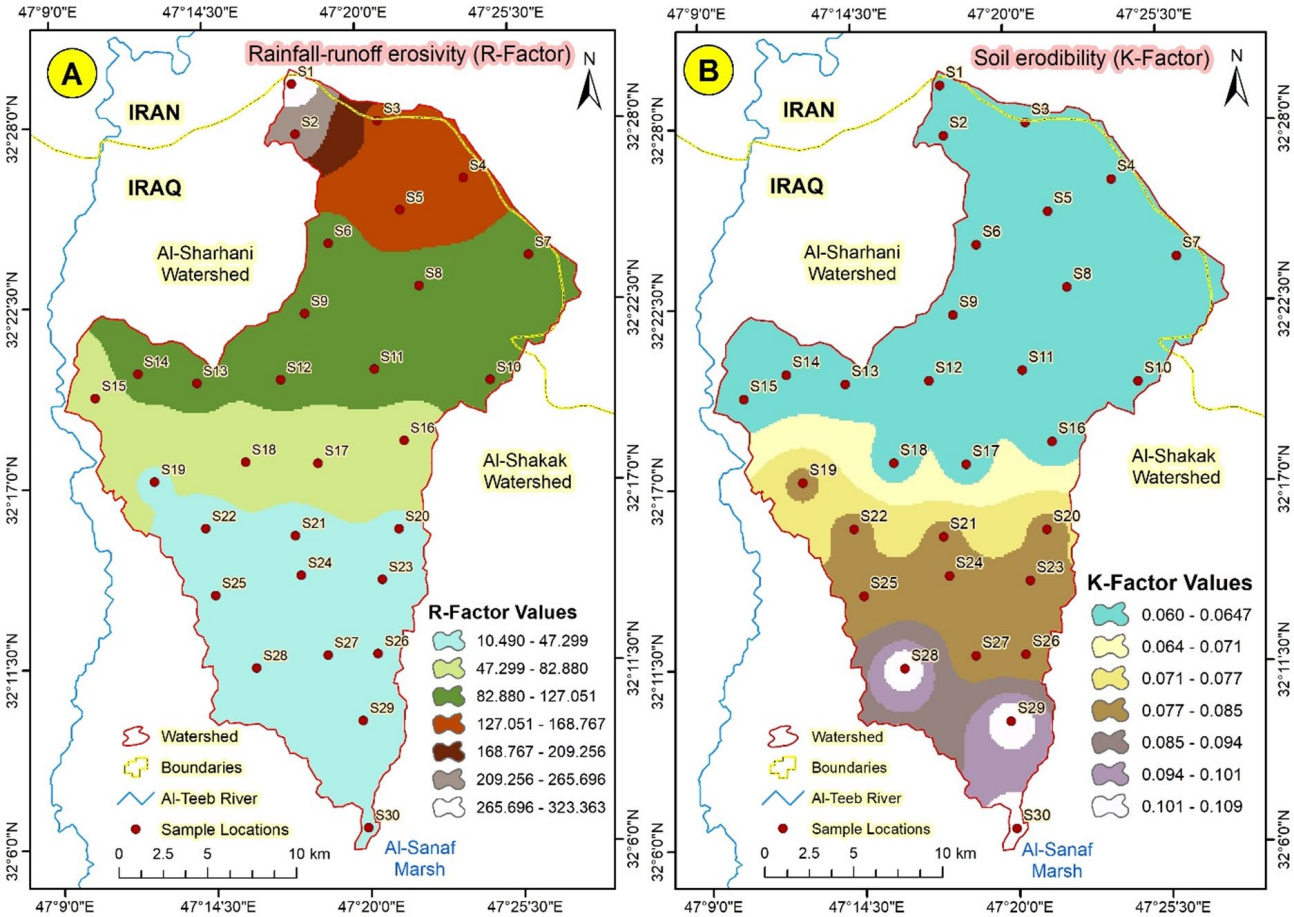
(**A**) Rainfall-runoff erosivity (R-factor) and (**B**) Soil erodibility (K-factor) in Abu-Ghraibat watershed.

Numerous studies demonstrated that the soil erosion rate in the catchment is highly responsive to rainfall^6,46^. Daily rainfall serves as a superior indicator of fluctuations in soil erosion rates, effectively characterizing the seasonal distribution of sediment output^32^. The benefits of utilizing yearly rainfall encompass its accessibility, simplicity of calculation, and enhanced regional uniformity of the exponent^47^. Consequently, in this analysis, the average yearly rainfall (calculated by dividing total rain by the number of rainy days) was utilized for the R-factor computation (Eq. 2). The computed rainfall-runoff erosivity (R-factor) values vary from 323.4935 at SW1 to 10.70138 MJ mm ha⁻¹ hr⁻¹ yr⁻¹ at the lower site in SW3 (Table 2). The maximum rainfall erosivity value is recorded in the northern region of the Abu Ghraibat watershed, attributed to the elevated terrain, which results in larger drop sizes, comparatively greater precipitation, and steep gradients (Fig. 13a). Rainfall erosivity progressively diminishes from the watershed’s northern to its southern regions. The south region of the watershed necessitates soil protection owing to elevated rainfall levels, in contrast to the north region.

#### Soil erodibility (K-factor)

The Soil Erodibility factor (K) denotes the vulnerability of soil or surface material to erosion, the transport capacity of sediment, and the volume and velocity of runoff resulting from specific rainfall input, as assessed under standardized conditions^48^. The standard condition is a unit plot measuring 22.6 m long, featuring a 9% gradient, kept in continuous fallow and cultivated up-and-down along the hill slope. The soil erodibility factor K was assessed based on soil textures. The K factor indicates the soil or surface material’s ability to resist erosion, the ease of sediment movement, and the volume and rate of runoff resulting from a specific rainfall input, as determined under typical conditions^49^. The K factor is influenced by particle size distribution, organic matter composition, structure, and permeability^50^. K values indicate the soil erosion rate per unit rainfall, as represented by the Runoff Erosivity (R) index (Fig. 13b). The soil erodibility factors (K) presented in Eq. (3) are most accurately derived from direct measurements conducted on natural runoff plots. A nomograph is typically used to determine the K factor for soil, depending on its texture, percentage of silt plus wonderful sand, percentage of sand, percentage of organic matter, soil structure, and permeability^12^.

#### The topographic factor (L.S)

The LS factor indicates a specific location’s susceptibility to topographic erosion^32^. This study confirmed that the LS factor is a primary and sensitive determinant of soil erosion, with the Abu-Ghraibat Watershed in the north identified as the principal physiographic unit. The steepness of a slope quantifies its influence on soil erosion rate. The terrain gradient has a significantly greater impact on soil erosion than the slope length. Table 2 indicates that roughly 30% of the territory has a very low slope, whereas moderately steep and very steep slopes characterize 60% of the territory. The northern, eastern, and northwestern regions of Abu-Ghraibat exhibit minimal vulnerability to soil erosion, characterized by LS factor values below 0.16 (Map 8). The topographic factor (L.S) denotes the impact of slope length and steepness on the erosion process. The LS factor was computed using flow accumulation and slope percentage as inputs. The results indicate that the topographic factor value increases from 0.108 to 0.127 as flow accumulation and slope increase.

#### Crop management factor (C)

**Fig. 14:**
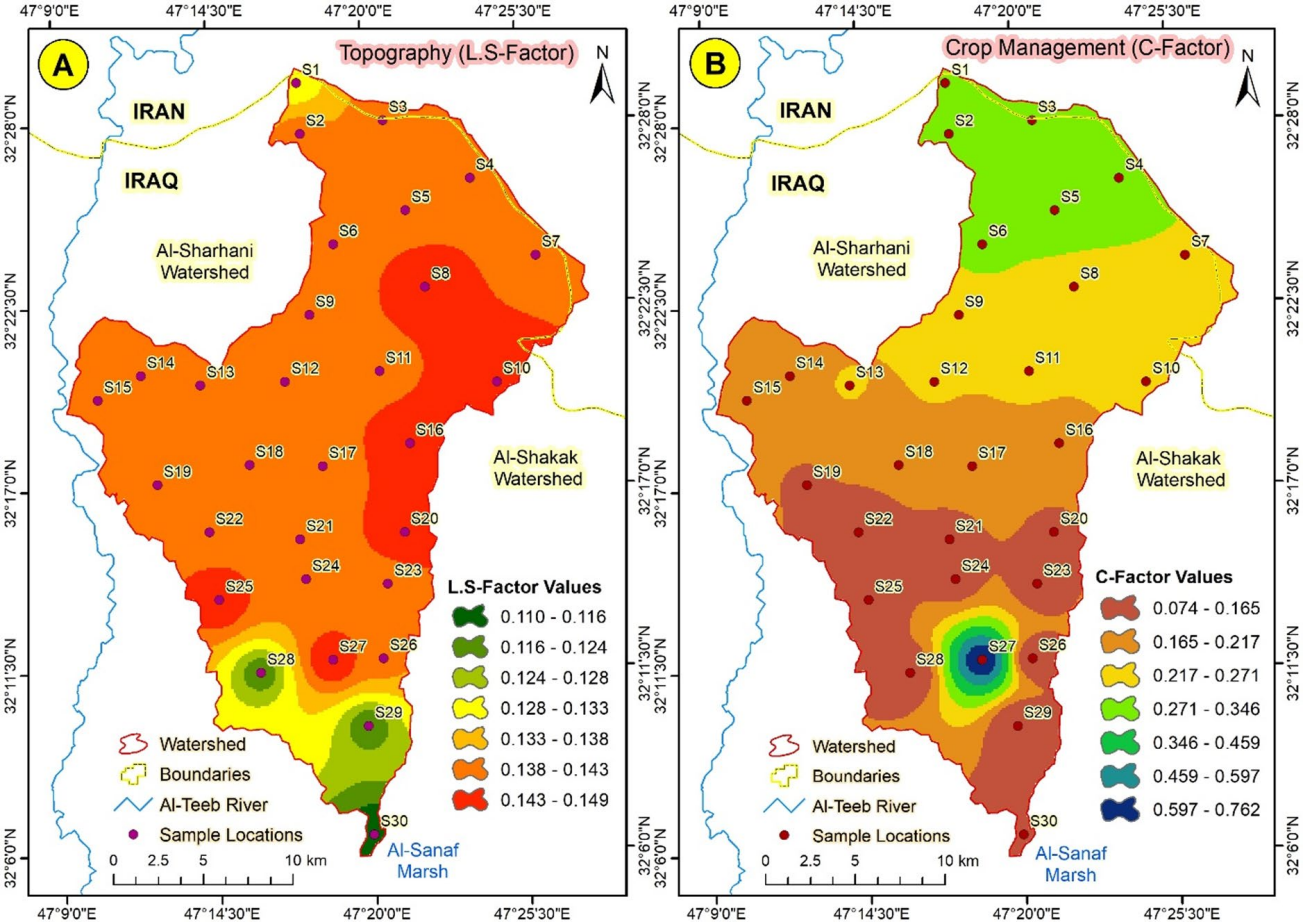
(**A**) The topographic factor (L.S), and (**B**) Crop Management Factor (**C**) in Abu-Ghraibat watershed.

The C factor denotes the land’s condition regarding vegetation density. Elevated C factor values indicate a higher likelihood of soil erosion, as they correspond to areas with minimal vegetation cover. The spatial distribution of the C factor in the study area ranged between 0.074 and 0.326. The comparative impact of management decisions can be directly associated with variations in the C factor, which ranged from approximately 0 for well-vegetated land to 1.000 for desolate or bare areas. Approximately 60% of the land exhibits diminished green cover, rendering it more susceptible to soil erosion. Conversely, 40% of the entire region (554.751 km²) is moderately to highly susceptible to soil erosion. Crop management factor (C) ranged between 0.074 and 0.326, with the lower values concentrated in the lower regions of the subwatershed3 and the higher values recorded in the upper areas of the subwatershed1 (Fig. 14).

#### Conservation practices factor (P)

The P factor is the ratio of soil erosion linked to certain support practices compared to the corresponding loss due to upslope management^32^. The P factor denotes the influence of particular soil management practices, including contour cultivation, strip cropping, terrace cultivation, and subsurface drainage. The research region’s land use and cover were uniform across all areas of the Abu-Ghraibat watershed, as they were subject to the same practices and land use.

#### Computed Spatial average soil loss and Temporal average soil loss per unit of area (A)

**Fig. 15:**
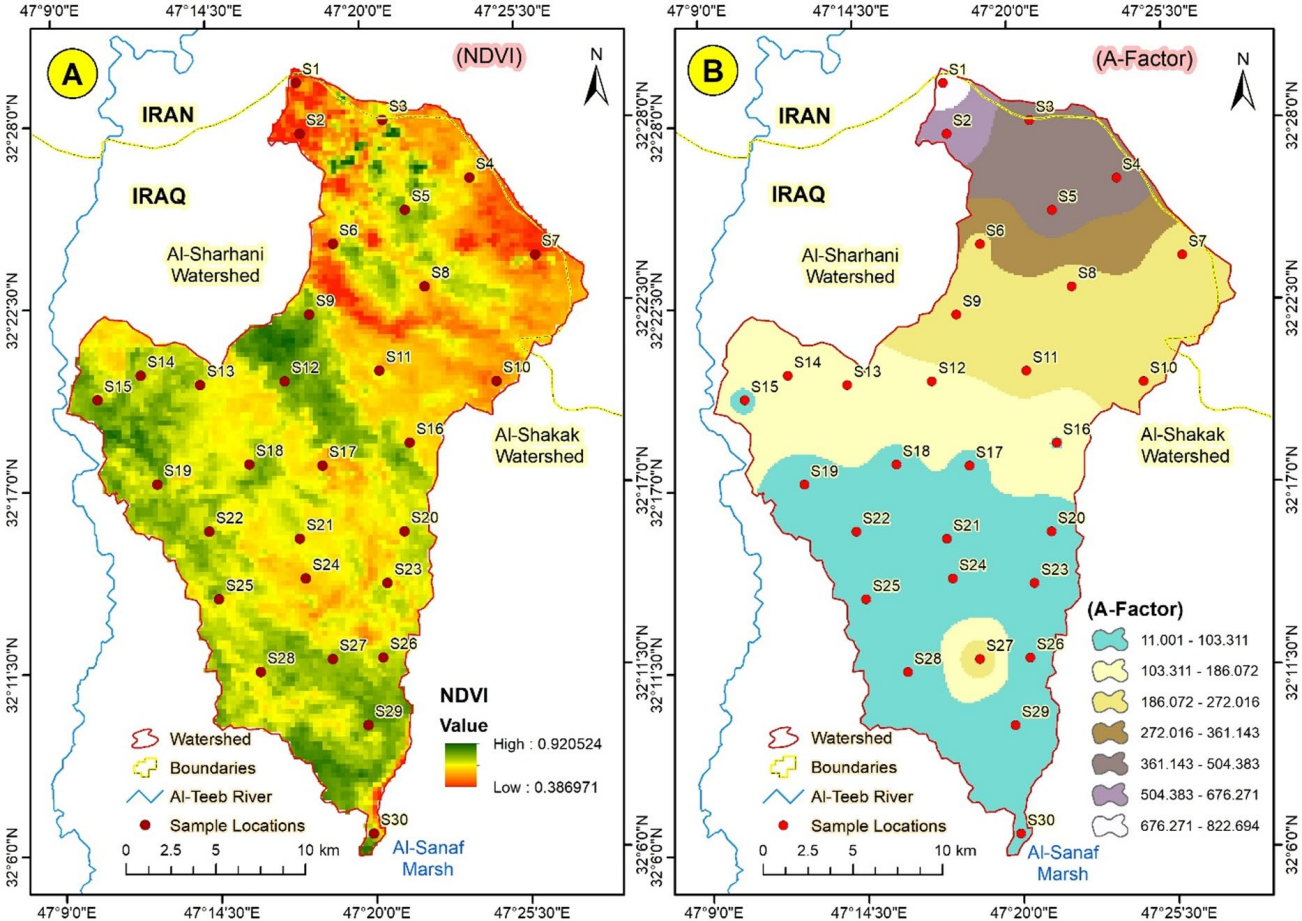
(**A**) The Normalised Difference Vegetation Index (NDVI), and (**B**) Computed spatial average soil loss and temporal average soil loss per unit of area (**A**) in Abu-Ghraibat watershed.

Table 1 showed that the Computed spatial average soil loss and temporal average soil loss per unit of area (A) values ranged between 11 and 823 t/yr, with the higher quantity of soil loss recorded in the upper regions of the Abu-Ghraibat watershed and the lower amount recorded in the lower areas. The soil erosion modulus increases markedly with slope, initially rising and then declining, as previously reported by^4,40^. Nevertheless, after the slope approached the threshold, soil erosion diminished. Specifically, we observed that erosion decreased at a slope of 35°, consistent with the findings of^51^. As the slope increases, soil erosion intensity progressively escalates, with average erosion intensity in areas of mild slope (≤ 25◦) lower than that in the entire gully, underscoring the significant impact of topography on soil erosion (Fig. 15). Slopes with gradients of 15° to 45° account for 85% of erosion, the primary cause of soil erosion in the southern sites of the Abu-Ghraibat watershed.

## Conclusions

This study calculated soil erosion in the Abu-Ghraibat watershed using the Revised Universal Soil Loss Equation (RUSLE), Geographic Information Systems (GIS), and Remote Sensing models, revealing that it resulted from three interconnected processes. In climate change, identifying vegetation coverage and slope thresholds for various land-use/land-cover classes is crucial for effective planning of vegetation restoration and of soil properties (organic matter, soil structure, soil permeability, and particle size distribution). Nonetheless, these thresholds may be influenced by geographical (local, watershed, and regional) and temporal scales, which affect the efficacy of soil erosion control—a vital landscape function. While it is true that soil erosion can affect land use, it is also well established that no area experiences erosion if it has sufficient vegetation cover. Identifying and distributing susceptible lands, categorized by varying degrees and intensities of degradation, should guide managers and policymakers in enhancing environmental, social, and economic conditions to mitigate the risk of land degradation substantially. Given the complexities of soil degradation, achieving the Land Degradation Neutrality goal by 2040 necessitates collaboration among scientists, governments, and managers. They must identify the primary factors contributing to soil degradation and erosion to promote effective governance for soil sustainability. Consequently, effective land degradation neutrality strategies must enhance the preservation of the quality and quantity of soil that underpin landscape services, including food and materials, as well as the frequently neglected regulating and supporting services that are essential for provisioning these services. To improve the accuracy of RUSLE-based soil loss estimates, we recommend that future studies conduct detailed field surveys of part of the Abu Ghraibat watershed over two consecutive years. These surveys will generate contour maps that reflect actual changes in surface morphology. By comparing the two contour maps, it will be possible to determine observed soil loss values (real A), which can then be used to validate and refine the RUSLE-predicted A values. This verification process will significantly enhance the reliability of soil erosion modelling and provide stronger guidance for soil conservation planning in semi-arid watersheds.

## Data Availability

Data is available by contacting the corresponding author.
